# Validation of the German version of the work and social adjustment scale in a sample of depressed patients

**DOI:** 10.1186/s12913-021-06622-x

**Published:** 2021-06-21

**Authors:** A. Heissel, J. Bollmann, M. Kangas, K. Abdulla, M. Rapp, A. Sanchez

**Affiliations:** 1 Department of Sport and Health Sciences, Intra-faculty Cognition Sciences, Faculty of Human Science, and Faculty of Health Sciences Brandenburg, Research Area Services Research and e-Health, University of Potsdam, Potsdam, Germany; 2 Social and Preventive Medicine, Department of Sports and Health Sciences, University of Potsdam, Potsdam, Germany; 3 Maria Kangas, Department of Psychology, Centre for Emotional Health, Macquarie University, Sydney, 2109 Australia

**Keywords:** Workability, Social functioning, Depression, Psychometric evaluation, Translation

## Abstract

**Background:**

Depression is one of the key factors contributing to difficulties in one’s ability to work, and serves as one of the major reasons why employees apply for psychotherapy and receive insurance subsidization of treatments. Hence, an increasing and growing number of studies rely on workability assessment scales as their primary outcome measure. The Work and Social Assessment Scale (WSAS) has been documented as one of the most psychometrically reliable and valid tools especially developed to assess workability and social functioning in patients with mental health problems. Yet, the application of the WSAS in Germany has been limited due to the paucity of a valid questionnaire in the German language. The objective of the present study was to translate the WSAS, as a brief and easy administrable tool into German and test its psychometric properties in a sample of adults with depression.

**Methods:**

Two hundred seventy-seven patients (*M* = 48.3 years, *SD* = 11.1) with mild to moderately severe depression were recruited. A multistep translation from English into the German language was performed and the factorial validity, criterion validity, convergent validity, discriminant validity, internal consistency, and floor and ceiling effects were examined.

**Results:**

The confirmatory factor analysis results confirmed the one-factor structure of the WSAS. Significant correlations with the WHODAS 2–0 questionnaire, a measure of functionality, demonstrated good convergent validity. Significant correlations with depression and quality of life demonstrated good criterion validity. The WSAS also demonstrated strong internal consistency (α = .89), and the absence of floor and ceiling effects indicated good sensitivity of the instrument.

**Conclusions:**

The results of the present study demonstrated that the German version of the WSAS has good psychometric properties comparable to other international versions of this scale. The findings recommend a global assessment of psychosocial functioning with the sum score of the WSAS.

**Trial registration:**

ISRCTN identifier: ISRCTN28972230. Date of registration June 26th 2018.

**Supplementary Information:**

The online version contains supplementary material available at 10.1186/s12913-021-06622-x.

## Background

Work disability has traditionally been associated with physical impairment, but with most jobs now being predominantly sedentary, office and desk jobs, physical impairment is only one component of disability. Over the past decade, psychiatric disabilities account for a significantly large portion of long-term disability claims and financial costs in Germany [1]. Since mental disorders have multimodal dimensions, it is essential to include the diverse impact of disease burden when evaluating health policy and planning health interventions. An operating social health system depends on patients’ social functioning and their ability to carry out the routine activities necessary to fulfill their social roles. Thus, to represent patients’ needs and the personal burden of disease, assessing the impairment caused by a mental disorder involves more than merely assessing the disease severity through disease-specific symptoms [2, 3]. Rather, the dimensions of workability and social functioning should also be addressed and considered in order to evaluate the multifaceted changes in one’s work function.

A person is generally considered to have workability if she or he possesses the physical, mental, and social health as well as standard basic competence essential for performing the tasks that most people in the same age group and sex would typically be able to accomplish [4]. Social adjustment, on the other hand, is commonly defined as the interchange between the individual and the social environment, in which the individual’s societal roles are accepted as appropriate or perceived in terms of the way the role performance conforms to the norms of the reference group [5]. In an early review, Anthony and Jansen (1984) [6] showed that there is at best little correlation between a person’s disease symptomatology and future work performance. This finding was explained by the fact that people may be ill but not necessarily show work impairment, while at the same time improvement of disease severity may not necessarily improve work functioning. Recent studies confirm this historic finding and show that instead, psycho-social functioning, regardless of the symptom severity, is a significant indicator of employment status [7] and that improvement in this dimension provides a clinically significant prediction of long-term symptom remission [8].

In Germany, the majority of long-term disability claims and financial costs are caused by impairments due to depression [9]. The range of work-related problems in depressive disorders includes temporary deficits due to loss of energy and decreased ability to concentrate, as well as a decrease in work performance due to more long-term cognitive, affective, and interpersonal dysfunction, repeated sick leaves, occupational disability, etc. Notably, limitations in workability is a major reason why employees apply for psychotherapy and receive insurance subsidies for treatments [10]. This reflects the increasing volume of recent studies utilising workability assessments as primary outcomes, and it is becoming increasingly accepted that reducing patients’ work disability is as important as improving symptom severity [11].

Although Anthony and Jansen (1984) [6] reported a lack of adequate tools to measure work functioning, over the past three decades an increasing number of scales have been developed to fill this vacuum. For the English-speaking population, a frequently used tool is the Work and Social Adjustment Scale (WSAS) [12, 13]. The WSAS is a self-report scale that measures the individual’s perception of work and social functioning and provides an assessment of the perceived ability to cope with mental health symptoms. It has been proven as a psychometrically reliable and valid questionnaire for comparing results among patients with various mental health disorders in a variety of settings [13, 14]. Different from other instruments for measuring work functioning, the WSAS was directly developed for the assessment of workability and social functioning in patients with mental health problems. It is designed to measure functional impairment attributable to an identified problem or disorder [13]. As it is short, easy to understand and fast to complete (M = 1.5 min., SD = 1.3) [15], its use both for assessment and for treatment evaluation is recommended [11].

With the increasing number of multinational research projects, the need to adapt health status measures for the use in languages other than the source language has grown. For the German language, to the best of our knowledge, there is no adapted and validated version of the WSAS to date. Although only one study made use of a German translation of the WSAS [16], the researchers did not investigate the psychometric properties of this instrument. In order to use this internationally validated instrument among the German population (comprising over 83 million individuals, of which approximately 69 million are of adult working age), and thus obtain internationally comparable results, it is timely to adapt and test the psychometric properties of the WSAS in the German language.

### Aim of the study

The primary aim of this study was to adapt and psychometrically validate the German version of the WSAS in a sample of adult patients with mild to moderate depression. A multistep translation and a subsequent validation were performed. The factorial validity of the scale was examined. It was hypothesized that a one-factor structure would explain the data model adequately. A multigroup CFA was tested to see whether measurement invariance across patients with a different severity of depressive symptoms (minimal/mild vs. moderate/severe) could be verified. It was hypothesized that the one-factor structure would demonstrate an acceptable to good fit for both subsamples and that measurement invariance could be established. Internal consistency, convergent validity, criterion validity, discriminant validity and floor and ceiling effects were specifically examined. A Cronbach’s alpha score above .70, high convergent validity, high criterion validity, and low floor and ceiling effects were expected.

## Methods

### Study design

The data for this validation study originate from the baseline assessment of the project “STEP.De -Sports Therapy for Depression”, which assessed the implementation of sports therapy as a non-inferior treatment alternative in depressed patients compared to psychotherapy [17]. The study was approved by the local ethics committee of the University of Potsdam (No. 17/2018) and the Freie Universität Berlin (No. 206/18) and was conducted in compliance with the Declaration of Helsinki. All methods were carried out in accordance with relevant guidelines and regulations. The study was registered in the ISRCTN registry (ISRCTN28972230).

### Procedure

Patients diagnosed with any of the inclusion diagnoses (F32.0, F32.1 (mild or moderate depressive episode), F33.0, F33.1 (recurrent depressive disorder, current episode mild or moderate), F34.1 (dysthymia), F43.2 (adjustment disorders), F43.8, F43.9 (reaction to severe stress), F48.0 (neurasthenia) and F41.2 (mixed anxiety and depressive disorder)) according to a general practitioner, in combination with an existing incapacity for work, were recruited by health insurance data managers in Berlin (Germany) between August 2018 and October 2020). To include patients with diverse social backgrounds, the patient sample was recruited from diverse urban districts. Patients were informed about the study aims and the voluntary nature of the study. When general interest was expressed, participants met a trained study assessor for patient education, regarding being informed of the data protection policy and to sign the informed consent forms. Via an electronic case reporting form (eCRF), participants provided their data and completed the WSAS and further self-report questionnaires outlined below (see Heissel et al., 2020 [17] for full details).

### Measures

Participants’ self-reported sociodemographic data on age, sex, education level, living status, income, first language, and employment were collected. For the education level, a variable with the three categories of low (lower secondary school), middle (secondary school diploma), and high education (university entrance qualification and university degree) levels was created. The income variable was categorised into low (< 1000€), middle (1000–2000€), and high (> 2000€) personal monthly net income.

### Work and social adjustment scale (WSAS)

The WSAS comprises 5 items (work, home management, social leisure, private leisure, and relationships), each rated on a 9-point Likert scale from 0 “not at all impaired” to 8 “very severely impaired” as a patient-reported outcome, which can also be pooled. The total score ranges from 0 to 40, with higher scores denoting higher levels of disability [12, 13]. Scores above 20 indicate moderately severe or worse impairment, scores between 10 and 20 represent significant functional impairment, and scores below 10 are considered subclinical [13]. The initial translation from English to German (forward translation) was performed by two independent German native speakers fluent in English. The resulting two German versions were synthesized, and technically and linguistically revised by a third German native speaker. The result was then translated back into the source language by an English native speaker fluent in German, but blind to the original WSAS (back translation). Non-equivalent translations were discussed until all translators agreed upon a functionally equivalent German version – ASAS: “Arbeits- und Sozialanpassungsskala”. The clinical guideline for cultural translation and adaptation of self-report scores was strictly followed in the translation process [18]. The result of this translation is presented in the Additional file 1 (Table S1). Participants were administered this measure online at baseline (prior to taking part in the treatment trial).

### World Health Organization Disability Assessment Schedule (WHODAS 2.0)

Within 5 days of the initial online assessment, an equivalent instrument for measuring workability, i.e. the World Health Organization Disability Assessment Schedule (WHODAS 2.0) [19], was administered by telephone through trained assessors, as this measure assesses functionality in multiple domains which is comparable to the WSAS. The WHODAS 2.0 is a questionnaire that assesses an individual’s level of functioning in six domains: cognition, mobility, self-care, getting along, life activities, and participation in society. In this study, the German 12-item screening version of WHODAS 2.0 [20] was used. For each item, respondents had to indicate the level of difficulty experienced during the previous 30 days using a five-point Likert scale from 1 “none” to 5 “extreme/cannot do”. The total score for global disability ranges from 0 “no disability” to 60 “complete disability”. The reliability of the WHODAS 2.0 in the present sample had a Cronbach’s α = .77.

### Beck depression inventory II (BDI-II)

Depressive symptoms were assessed by the German version [21] of the Beck Depression Inventory II (BDI-II) [22]. The BDI-II is a 21-item self-report depression screening measure. Individuals were asked to respond to each question based on a two-week time period. Items were rated on a four-point Likert scale ranging from 0 to 3. The maximum total score is 63, with higher scores indicating higher levels of depressive symptoms. According to the BDI-II manual [22], a score of 0–13 indicates minimal depression, 14–19 mild depression, 20–28 moderate depression, and 29–63 severe depression. The reliability in the present sample had a Cronbach’s α = .91.

### Single item general impairment

For validity measures, a single-item question was developed from the WSAS (“Meine Depression beeinträchtigt mich im Alltag/ in der Freizeit/ im Berufsleben – My depression affects me in everyday life/ in my free time/ in my work life”) to assess the global impairment due to depression. Participants rated the item on a Likert scale from 0 “not at all impaired” to 8 “very severely impaired” with every second step marked, so that higher values indicated greater impairment.

### 12-item short form survey (SF-12)

To assess health-related quality of life, the 12-Item Short Form Survey (SF-12) [23, 24] questionnaire was used. It consists of seven questions including 12 items and representing eight domains. Next to a weighted sum score, items can be grouped into two subscales, the mental component summary (MCS-12) and the physical component summary (PCS-12). The PCS-12 represents four domains, namely general health perception, physical functioning, physical role functioning, and pain. The MCS-12 reflects the four domains of emotional role functioning, mental well-being, negative affectivity, and social functioning. Both summary scores range from 0 to 100, with higher scores indicating better quality of life. In this study, only the two SF-12 sub-summary scores were used to better differentiate between the mental and physical constructs of workability.

### Data analyses

Data analyses were performed in SPSS (IBM Corp. Released 2017. IBM SPSS Statistics for Windows, Version 25.0.0.1 Armonk, NY: IBM Corp.) and R Studio (version 1.2.5042 for Macintosh). For all analyses, statistical significance was set at a *p* value of less than .05.

Sample characteristics were summarized as frequencies and percentages for the categorical variables and as means and standard deviations (SD) for the continuous variables. The Shapiro-Wilk test was used to assess normality. The non-parametric Kruskal Wallis and Mann-Whitney U test were used to investigate the differences in the WSAS total score by age, sex, income and education level. Correlation between the continuous variable of age and the WSAS total score was also examined. Chi-square tests were used to investigate the differences in the level of impairment (WSAS score < 10, subclinical impairment;10–20, significant functional impairment; > 20 moderately severe or worse impairment) by sex, income, and education level.

#### Floor and ceiling effects

To examine the usability of the WSAS in a homogeneous group of patients with mild to moderately severe depressive disorders, floor and ceiling effects were examined by evaluating the means and standard deviation of each item and testing these against the lowest and highest possible scale values via one sample t-test. Furthermore, the frequency of participants with the lowest and highest possible scores and the skew distribution for each item were assessed. The cutoff for a significant floor or ceiling effect was set at ≤ 15% [25]. For the skewness distribution, values less than − 1 or greater than + 1 were considered highly skewed, values between − 1 and − .50 or between + .50 and + 1 were considered moderately skewed, and values between − .50 and + .50 were considered approximately symmetrical [26].

#### Factorial validity

To test the factorial validity, Confirmatory Factor Analysis (CFA) was performed using the Lavaan package in R Studio [27]. To test the hypothesis that the WSAS is best interpreted as a one-factor structure, two models were tested: the original one-factor model (Model 1, M1), and a second model (Model 2, M2) with a post-hoc modification that allowed items 3 and 5 to correlate, as indicated by the modification indices (M.I.).

Models were evaluated using a chi-square test and additional fit indices. As the chi-square is known to be affected by the sample size, a relative/normed chi-square (ratio of the chi-square test to the degrees of freedom) [28] that minimizes the impact of sample size on the model fit was calculated. A value < 2 for the normed chi-square is considered a good model fit, and a value < 3 an acceptable model fit [29]. The Bentler Comparative Fit Index (CFI) and the Tucker-Lewis Index (TLI) were used as comparative fit indices. Following the literature, an acceptable model fit was set by values ≥ .90, and values ≥ .95 indicated a good model fit [30]. The standardized root-mean-square residual (SRMR) and root-mean-square error of approximation (RMSEA) were assessed as absolute fit indices. For the SRMR, values < .05 were considered good and values < .10 were considered acceptable [31]. For the RMSEA values < .05 were interpreted as good, and values between .05 and .08 as acceptable [32, 33]. Modification indices were calculated to identify where linear constraints might be relaxed to improve model fit [34]. To ensure that the characteristics of the dataset were suitable for CFA to be conducted on the study sample, the linear relationship between WSAS items was graphically validated by Q-Q plots. Multifactorial normal distribution was tested by a Shapiro-Wilk test and a Kolmogorow-Smirnow test. As these tests did not confirm normal distribution, the maximum likelihood estimation with robust (Huber-White) standard errors and a scaled test statistic (Yuan-Bentler) was used for CFA. To compare the two different models, the Satorra-Bentler Scaled chi-square difference test (SBS-χ^2^) was used [35], where the usual normal-theory chi-square statistic is divided by a scaling correction to better approximate the chi-square under non-normality. Because the SBS-χ^2^, as the chi-square test used to test goodness of fit, is sensitive to sample size [36], the difference in CFI (∆CFI) [37] and two predictive fit indexes, the Akaike’s information criterion (AIC) values [38] and the Bayesian Information Criterion (BIC) [39], were also considered. A decrease of less than .01 in the fit of the more parsimonious model on the CFI should be treated as support for that model [37, 40]. Lower BIC and AIC values indicate better model fit [31]. A difference of 10 points between models was accepted as a relevant difference.

#### Measurement invariance

To examine whether the WSAS had the same psychometric properties across patients with a different severity of depressive symptoms (minimal/mild vs. moderate/severe) according to the BDI-II  [22], measurement invariance of Model 2 was tested in a series of multigroup CFA with three levels of invariance (configural, weak, and strong invariance) [40]. Whereas configural invariance imposes the same factor structure in all groups, weak invariance constrains all factor loadings to be equal across groups and strong invariance additionally constrains the equality of intercepts. All model fits were tested using robust maximum likelihood (robust ML) and full information maximum likelihood (FIML) estimation.

Model comparisons were processed using Satorra-Bentler scaled chi-square difference test (ΔSBSχ^2^) for two nested models [41], changes in fit indices, and AIC and BIC values. For testing weak invariance, a change of ≥ − .01 in CFI, supplemented by a change of ≥ .03 in SRMR or a change of ≥ .015 in RMSEA would indicate non-invariance. For testing strong invariance, a change of ≥ − .01 in CFI, supplemented by a change of ≥ .015 in SRMR or a change of ≥ .015 in RMSEA would indicate non-invariance. Among the three indexes, CFI is chosen as the main criterion [40].

#### Internal consistency

For reliability measures, internal consistency tested by Cronbach’s alpha (α) [42] and the coefficient omega (ω) [43, 44] were assessed. Coefficients Cronbach’s alpha and omega above .70 were considered satisfactory [45, 46].

#### Convergent validity

Convergent validity was assessed by the correlations between the WSAS and WHODAS 2.0, as well as their individual items, as a different measure of workability and social functioning. As Shapiro-Wilk test scores for WSAS and WHODAS 2.0 scores did not follow the normal distribution (*p < .*05), the non-parametric coefficient of Spearman’s rho was used for the correlations between the WSAS and the other instruments.

#### Criterion validity

To examine the criterion validity, Spearman rho correlation coefficients between the WSAS and related constructs were calculated. It was determined by the correlation between the WSAS and the BDI-II as a measurement of symptom severity, between the WSAS and the Single Item as a measure of General Impairment, and between the WSAS and the two SF-12 subscales as a measurement of mental and physical health status. Correlations less than .30 were considered weak, correlations between .30 and .49 were considered moderate, and correlations greater than .49 were considered strong [47].

#### Discriminant validity

Discriminant validity was evaluated using Kruskal Wallis test to investigate the differences in the WSAS total score among four groups of patients with a different severity of depressive symptoms according to the BDI-II (minimal, mild, moderate and severe).

## Results

The sample comprised *n* = 277 (72.7% women) patients with mild to moderately severe depression (Beck Depression Inventory II (BDI-II) mean score = 22.28, *SD* = 10.14). The mean age was *M* = 48.3 years (*SD* = 11.1, range 20–65) and 80.9% of the participants had worked within the last 3 months. Further patient characteristics are shown in Table 1.

**Table 1:** Characteristics of the sample (*n* = 277)

	n	No. (%), range
Age (years), M (SD) range	275	48.3 (11.0), 20–65
Sex	275	
Female		200 (72.7)
Male		75 (27.3)
Education level	266	
Lower secondary school		22 (8.3)
Secondary school		161 (58.1)
Higher education		83 (31.2)
Living status	274	
Alone		70 (25.5)
Not alone		204 (75.5)
Personal monthly net income	262	
Low		28 (10.7)
Middle		158 (60.3)
High		76 (29.0)
First language	263	
German		255 (97.0)
Other		8 (3.0)
Worked within the last 3 months	272	
Yes		220 (80.9)
No		52 (19.1)
Depressive symptoms (BDI-II)	277	
Minimal		54 (19.5)
Mild		62 (22.4)
Moderate		81 (29.2)
Severe		80 (28.9)

*BDI*-*II* Beck Depression Inventory II

### Descriptives of the WSAS

The mean WSAS total score in the present sample was 18.55 (*SD* = 10.17, range 0 to 40). As responses were obligatory in the eCRF, there were no missing values for WSAS items. The lowest mean score was found for item 5 (impairment in forming and maintaining close relationships) with a value of 3.01 (*SD* = 2.35), and the highest value was for the work item (item 1; *M* = 4.60, *SD* = 2.74). The WSAS mean overall value per item was 3.71 (*SD* = 2.03) on scales ranging from 0 to 8. As for the level of impairment according to the WSAS total score, 44.8% of the patients reported moderately severe or worse impairment, 33.6% reported significant functional impairment and 21.7% reported subclinical impairment.

No statistically significant differences in the WSAS total score within the items of age, sex education level or income were found in the sample, nor differences in the level of impairment for sex or education level. Significant differences were found in the level of impairment between income groups (*p* = .035), with a higher percentage of patients with moderately severe or worse impairment reporting low- or middle-income levels.

### Floor and ceiling effects

The percentage of participants answering with the lowest or highest possible value illustrates the skewness of the item distribution. For items 2 and 5, 15.9 and 17.0% of the participants answered with the lowest possible value of 0. For item 1, 23.8% of participants answered with the highest possible value of 8. The percentage of participants answering with the lowest or highest value was less than 15% for the rest of the items.

When testing every item’s mean value against both the highest possible scale value of 8 and the lowest possible value of 0 in one-sample, one-tailed t-tests, each test showed a significant difference (all *p < .*001), indicating the absence of floor and ceiling effects. The skewness distribution of the individual items ranged from - .21 to .47 (all *SE* = .15), indicating that the distribution is approximately symmetric.

### Factorial validity

**Fig. 1:**
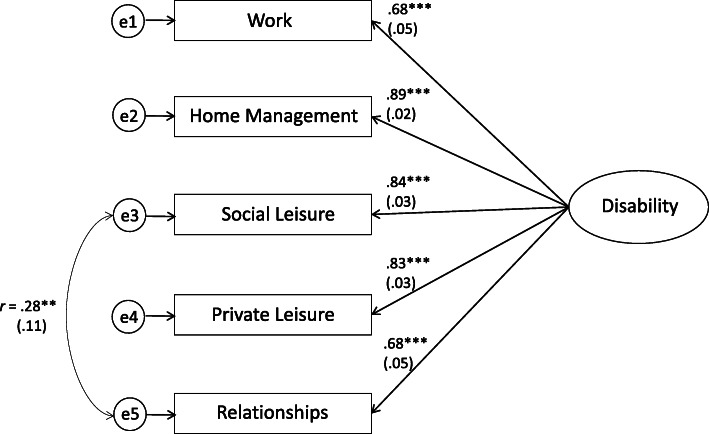
The one-factor CFA model of the WSAS. Note. The model includes residual item correlation between items 3 and 5 for impairment in social leisure activities and maintaining close relationships. Significant standardized parameter estimates (robust standard errors). **p < .01; ***p < .001

The chi-square test showed no perfect model fit (χ2 (5) = 20.386, *p =* .001), but most of the fit indices of the single-factor solution indicated an acceptable to good fit for the tested model (M1): CFI_M1_ = .974, TLI_M1_ = .947, SRMR_M1_ = .033, although the RMSEA value was above the acceptable value of .08 (RMSEA_M1_ = .170). As indicated by the modification indices (M.I. = 14.11), the theoretical assumption of conditional independence was relaxed, and a residual item correlation between items 3 and 5 (item correlation *r* = .70, *p* < .001) was included in the model (M2). From a theoretical perspective, this is a reasonable assumption, as it is these two items that ask about the impairment of social relatedness. This adjustment led to an improvement of the fit in all indices (CFI_M2_ = .987, TLI_M2_ = .969, SRMR_M2_ = .023, RMSEA_M2_ = .096). The goodness-of-fit indices for the two tested models are presented in Table S2 (in Additional file 1). When comparing the first (M1) and the second model (M2) a significantly better fit for the second model was found (ΔSBS-*χ*^*2*^ (1) = 5.869, *p* = .015), and the difference in practical fit between both models was meaningful (ΔCFI = .013). The lowest AIC (AIC_M1_ = 5615.171; AIC_M2_ = 5603.624) and BIC (BIC_M1_ = 5619.703; BIC_M2_ = 5608.609) values obtained for the second model (M2) also support this solution as the preferred model. Further theoretically embedded improvements were not indicated by the modification indices, and the second model (M2) was chosen as the final model. Figure 1 displays the fitted one-factor model for the WSAS with released residual correlation of items 3 and 5 (M2). All factor loadings were positive and substantial (*p* < .001) for the single-factor WSAS model. They ranged between *r* = .68 and .89, and thus were adequate for all items.

### Measurement invariance

When calculating the Model 2 for the two subsamples separately, most of the fit indices remained good (see Additional file 1 (Table S3)). As for the measurement invariance across the two subsamples, configural invariance, that imposes the same factor structure in all groups was established, as the fit indices met the generally accepted fit criteria. However, when testing the weak invariance (imposing equality of factor loadings) the constrained model differed significantly from the unconstrained model (ΔSBS-*χ2* (4) =15.03, *p* < .002), and the fit indices for the constrained model were substantially different from the unconstrained model (∆CFI > −.01, ∆SRMR = .03, ∆RMSEA > .015). As configural invariance could not be established, no further analyses were conducted.

### Internal consistency and item analyses

The internal consistency of the WSAS total score was very good, with a Cronbach’s α = .89 (95% CI .85 to .91) and a coefficient ω = .88.

Furthermore, all items showed a good adjusted item-scale correlation (all *r* > .40). The work item (item 1) had the lowest correlations to all other WSAS items and to the total score. Item-total statistics showed a slight improvement for Cronbach’s α when deleting item 1 (α = .90), suggesting that item 1 and items 2 to 5 may be interpretable in different ways. However, this small improvement in internal consistency and the differences in item-item correlations do not contradict the finding of high internal reliability. Although the item-item correlations between all five items are all positive and significant, Table S3 (in Additional file 1) shows that certain item-item correlations are stronger than others.

### Convergent validity

To examine convergent validity, correlations between WSAS and WHODAS total scores are shown in Table 2. Overall correlation between the two scales was strong (*r*_*s*_ = .69, *p* < .001). In addition, moderate correlations were found between the WSAS total score and some of the individual WHODAS items: item 2 “Household” (*r*_s_ = .58, *p* < .001), item 3 “Community activities” (*r*_*s*_ = .53, *p* < .001) and item 5 “Emotionally affected” (*r*_*s*_ = .53, *p* < .001). Also, strong correlations were found between the WHODAS total score and all the individual WSAS items (all r_s_ > .50, *p* < .001). Matching correlations were also found for several items between the WSAS and WHODAS. All associations between individual items are displayed in Table S4 (Additional file 1).

**Table 2:** Correlations between WSAS, WHODAS, BDI-II, single-item general impairment, and SF-12 subscales mean scores

WSAS	18.55 (10.17)	1					
WHODAS	29.41 (8.95)	.69^**^	1				
BDI-II	22.28 (10.14)	.79^**^	.66^**^	1			
Single-Item General Impairment	4.64 (2.30)	.81^**^	.61^**^	.74^**^	1		
PCS-12	43.18 (8.89)	−.52^**^	−.53^**^	−.44^**^	−.41^**^	1	
MCS-12	31.86 (9.03)	−.63^**^	−.55^**^	−.68^**^	−.64^**^	.13^*^	1

Sample size ranged from *n* = 252 to *n* = 277 due to missing values in the answers

*WHODAS* World Health Organization Disability Assessment Schedule, *BDI-II* Beck’s Depression Inventory II, *PCS-12* 12-Item Short Form Health Survey Physical Composite Scale, *MCS-12* 12-Item Short Form Health Survey Mental Health Component Scale

*p < .05; **p < .001

### Criterion validity

Table 2 displays the Spearman rho correlations of individual mean scale scores for criterion validity. The WSAS total score showed strong positive correlations with the BDI-II total score (*r*_*s*_ = .79, *p* < .001) and the Single Item General Impairment (*r*_*s*_ = .81, *p* < .001). In addition, moderate negative associations between the WSAS total score and the PCS-12 (*r*_*s*_ = − .52, *p* < .001) and MCS-12 subscales were found (*r*_*s*_ = − .63, *p* < .001).

### Discriminant validity

The Kruskal-Wallis test showed significant differences in the WSAS total score across the four groups of patients with a different severity of depressive symptoms according to the BDI-II (*H* [3, 277] = 157.311 *p* < .001) (see Additional file 1 (Table S5)). As expected, post hoc pairwise comparisons showed that the group with severe depressive symptoms had a statistically significant higher WSAS total score than the groups with moderate, mild (*p* < .001) or minimal (*p* = .001) depressive symptoms. The group with moderate depressive symptoms had higher WSAS total scores than the groups with mild and minimal depressive symptoms (both *p* < .001). The group with mild depressive symptoms had higher WSAS total scores than the group with minimal depressive symptoms (*p* < .001).

## Discussion

The main aim of the current study was to perform a linguistic translation of the WSAS from English into the German language and analyze its psychometric properties in a sample of German-speaking patients with mild to moderately severe depression. Factorial analyses confirmed the one-factor model for this scale. The WSAS – German translated version displayed good psychometric properties, with satisfactory to very good internal consistency, convergent validity, criterion validity, discriminant validity and sensitivity without floor and ceiling effects.

The overall scale mean value was 18.55 (*SD* = 10.17), indicating significant functional impairment in the present sample [13], which is comparable to values found in other international studies with depressed patients [15, 48, 49]. Except for depression severity, there was no evidence that the WSAS total score was associated with sample characteristics of age, sex, net income or education level, thus confirming measurement sensitivity within a homogeneous group. One-tailed, single-sample t-tests resulted in significant differences between item mean values and both ceiling and floor values, indicating a reasonable sensitivity for the use in a homogenous sample who feel impaired in their everyday functioning due to depression.

The results of the confirmatory factor analyses strongly supported the mono-factorial structure of the WSAS, which is congruous with previous findings [14, 50, 51]. When calculating the one-factor structure separately for the subsamples with minimal/mild and moderate/severe depressive symptoms, most of the fit indices showed a good model fit to the data.

With a Cronbach’s alpha score of .89, internal consistency was found to be strong. This result is in line with findings from other studies, which used the WSAS among patients with various mental disorders [11, 14, 50, 52, 53].

One strength of the current study was the comparison, for the first time, of the self-administered assessment of psycho-social functioning with an interviewer-rated assessment of a convergent construct by using the WHODAS 2.0 questionnaire. In the present study matching correlations were found for several items between the WSAS and WHODAS, referring to both private and work life, indicating good convergent validity. The correlation between WSAS and WHODAS global scores is high, but lower than the association between the WSAS and symptom severity or general impairment. As the scores of the WHODAS and WSAS address impairment in social functioning, a stronger association between these scores might be expected, but could be a result of different approaches of the instruments, e.g. the measurement method, different metrics of the scales, or differences between survey methods and assessed time frames. Despite the differences, the correlation score of *r* = .69 is considered high and as a strong indication of convergent validity. The higher correlation of WSAS score (.79) than WHODAS score (.66) to symptom severity speaks for the clear embedding of the WSAS in the psychological background against which the WSAS was developed and helps to evaluate the implications of the depression for the psycho-social functioning. Due to its rapid administration and the short time period assessed, the WSAS can also be easily used in clinical settings and reflects the current perception of the patient.

The WSAS is also significantly positively correlated to a high degree with the single depression item, as well as symptom severity assessed by the BDI-II. This association in particular may be relevant to the construct validity of the WSAS, as depression itself is associated with increased disability. It is also in accordance with other studies that report strong correlations to symptom severity [8, 13, 50], although correlation in this study seems to be somewhat higher compared to other reports. In general, WSAS scores in studies with depressed patients seem to show slightly higher correlations to symptom severity when compared to studies of other mental health disorders. The high correlation with symptom severity reflects the clinical sample of depressed patients, while the question also arises whether the WSAS as a generic instrument should be used without mentioning the assessed disease but with a general phrase like “my problem” instead. Unfortunately, most published studies to date, do not report precisely how they implemented the WSAS. Furthermore, when a construct as broad as functional impairment is measured in a specific patient sample, in this case depression, the different aspects of this disease are clumped together and can shift the result in one specific direction [54].

Furthermore, a strong correlation between WSAS and PCS-12 shows that the physical aspect of work and household chores is present, whereas the higher correlations with the MCS-12 subscale and symptom severity in depression than in the physical dimensions indicate that it is possible the WSAS may measure a concept of disability which is more strongly associated with the capacity to participate in life than the physical demands of employment per se. Therefore, the use of the WSAS may be more appropriate for investigations specifically targeting a concept of disability relying less strictly on physical capacity and accounting more for the ability to be socially active.

The Kruskal-Wallis result showed good discrimination of WSAS scores between the four groups with different severity of depressive symptoms. The impairment level differed significantly between the groups with different severity of depressive symptoms, and higher impairment level was present at higher symptom severity levels, supporting a good criterion validity for the WSAS measures.

### Limitations

A few limitations of this study need to be noted when interpreting the results. First, the validation was conducted in a homogenous sample of patients with mild to moderately severe depression. Therefore, generalizability for different clinical and non-clinical samples has yet to be established. In addition, as only datasets from 277 participants were available for this study, the random sample was quite small. Further, we have reported the absence of floor and ceiling effects in the WSAS, since when testing every WSAS item’s mean value against both the highest possible scale value of 8 and the lowest possible value of 0 in one-tailed t-tests, each test showed a significant difference. However, it should also be noted that for item 1, 23.8% of participants answered with the highest possible value of 8, while for items 2 and 5, 15.9 and 17.0% of the patients answered with the lowest possible value of 0. Ceiling and flooring values by more than 15% of the participants are typically considered to be significant, as they compromise the capacity of an instrument to detect change [25].

The lack of conventional test-retest reliability in the present study is also a limitation that needs to be mentioned.

### Future research

The current results have shown that the use of the German version of the WSAS is reliable and valid in a patient sample with mild to moderately severe depression. To further validate its usefulness and generalizability, validation of the German version scale should be conducted in various other patient samples for different disorders, within other important (disorder-relevant) subgroups, while performing alternative assessment and treatment types, and across other settings. This would strengthen the understanding of its usefulness in German-speaking populations. Additionally, normative data for the general population could be a further improvement in interpreting WSAS values. To evaluate the validity of the WSAS for the use in clinical settings or as an intervention outcome, test-retest reliability and sensitivity to change should be tested with longitudinal data. To further expand the use of the WSAS, validation in other languages for a variety of other mental disorders should be a research focus. As it is a strength of this generic instrument, a strong background with norm data sets for further classification and comparison of social functional impairment across different psychological disorders is necessary.

## Conclusion

In this study, we translated and psychometrically evaluated the German version of the WSAS in a sample of depressed patients. The results demonstrated that it has very good internal consistency and a mono-factorial structure; it coherently measures the intended construct of work and social impairment. With its specific focus on impairments caused by a psychological disorder, it differentiates itself from other similar assessment tools that measure psychosocial functioning. The findings support the validity of the WSAS according to conventional standards, recommending the interpretation of a general WSAS score. Overall, the current study is the first to assess the psychometric properties of the German version of the WSAS and the results indicate that it is a valid and sensitive measure of impaired functioning, which provides readily interpretable comparisons to those in the English language.

## Supplementary Information

**Additional file 1: Table S1.** WSAS items from the original English version and the adapted German version. **Table S2.** Goodness-of-fit indices of the tested models. **Table S3.** Confirmatory factor analyses for subsamples with different severity of depressive symptoms and invariance testing. **Table S4.** Spearman rho correlations between WHODAS and WSAS items. **Table S5.** WSAS total score among patients with different severity of depressive symptoms.

## Data Availability

An anonymized dataset used and/or analyzed during the current study is available from the corresponding author on reasonable request.

## Abbreviations

AIC: Akaike information criterion
ASAS: Arbeits- und Sozialanpassungsskala
BIC: Bayesian information criteria
BDI-II: Beck’s Depression Inventory II
CFI: Bentler Comparative Fit Index
CFA: Confirmatory factor analysis
eCRF: Electronic case reporting form
MCS-12: 12-Item Short Form Health Survey Mental Health Component Scale
PRO: Patient-Reported Outcome
PCS-12: 12-Item Short Form Health Survey Physical Composite Scale
RMSEA: Root-mean-square error of approximation
SBS-χ^2^: Satorra-Bentler Scaled chi-square difference test
SF-12: 12-Item Short Form Survey
SRMR: Standardized root-mean-square residual
TLI: Tucker-Lewis Index
WHODAS: World Health Organization Disability Assessment Schedule
WSAS: Work and Social Adjustment Scale
