# Supplementary material for: Validation of the German version of the work and social adjustment scale in a sample of depressed patients
Source: BMC Health Serv Res. 2021 Jun 21;21:593. doi: 10.1186/s12913-021-06622-x (PMC8218495; doi:10.1186/s12913-021-06622-x)
Supplement: Supplementary file 1 — Additional file 1: Table S1. WSAS items from the original English version and the adapted German version. Table S2. Goodness-of-fit indices of the tested models. Table S3. Confirmatory factor analyses for subsamples with different severity of depressive symptoms and invariance testing. Table S4. Spearman rho correlations between WHODAS and WSAS items. Table S5. WSAS total score among patients with different severity of depressive symptoms. [file 12913_2021_6622_MOESM1_ESM.docx]

**Validation of the German Version of the Work and Social Adjustment Scale in a Sample of Depressed Patients**

Heissel, A., Bollmann, J., Kangas, M., Abula, K., Rapp, M., Sanchez, A.

| **Table S1** WSAS items from the original English version^a^ and the adapted German version | | |  |
| --- | --- | --- | --- |
| Item | English – Work and Social  Adjustment Scale (WSAS) | German – Arbeits- und Sozialanpassungsskala (ASAS) |  |
| 1 | Because of my [problem] my ability to work is impaired. ‘0’ means ‘not at all impaired’ and ‘8’ means very severely impaired to the point I can't work. | Wegen meiner Depression^b^ bin ich in meiner Arbeitsfähigkeit beeinträchtigt.  Dabei bedeutet '0' = Gar nicht beeinträchtigt und '8'= sehr stark beeinträchtigt bis zu dem Punkt, dass ich nicht arbeiten kann. |  |
| 2 | Because of my [problem] my home management (cleaning, tidying, shopping, cooking, looking after home or children, paying bills) is impaired. | Wegen meiner Depression bin ich in der Haushaltsführung (Putzen, Aufräumen, Einkaufen, Kochen, Kinder betreuen, Rechnungen bezahlen) beeinträchtigt. |  |
| 3 | Because of my [problem] my social leisure activities (with other people e.g. parties, bars, clubs, outings, visits, dating, home entertaining) are impaired. | Wegen meiner Depression bin ich in meinen sozialen Freizeitaktivitäten (mit anderen Leuten, z.B. Partys, Bars, Clubs, Ausflüge, Besuche, Verabredungen, Heimunterhaltung) beeinträchtigt. |  |
| 4 | Because of my [problem], my private leisure activities (done alone, such as reading, gardening, collecting, sewing, walking alone) are impaired. | Wegen meiner Depression bin ich in meinen privaten Freizeitaktivitäten (alleine ausgeübt, so wie Lesen, Gartenarbeit, Sammeln, Nähen, alleine Spazierengehen) beeinträchtigt. |  |
| 5 | Because of my [problem], my ability to form and maintain close relationships with others, including those I live with, is impaired. | Wegen meiner Depression bin ich in meiner Fähigkeit enge Beziehungen mit anderen einzugehen und aufrechtzuerhalten, inklusive der Personen, mit denen ich zusammenwohne, beeinträchtigt. | |
| ^a^Mundt et al. (2002)  ^b^Also included is the specific naming of “Depression” in the German version instead of “problem” in the English version. | | | |

| **Table S2** Goodness-of-fit indices of the tested models (N = 277) | | | | | | | | | |
| --- | --- | --- | --- | --- | --- | --- | --- | --- | --- |
|  | χ^2^ | *df* | χ^2^/*df* | CFI | TLI | SRMR | RMSEA (90%CI) | AIC | BIC |
| Model 1 | 20.386** | 5 | 4.08^n^ | .974^g^ | .947^a^ | .033^g^ | .124^n^ (.071-.183) | 5615.171 | 5619.703 |
| Model 2 | 12.760* | 4 | 3.19^n^ | .987^g^ | .969^g^ | .025^g^ | .096^n^ (.040-.157) | 5603.624 | 5608.609 |
| Note. *df* Degrees of Freedom, *CFI* Comparative Fit Index, *TLI* Tucker-Lewis Index, *SRMR* Standardized Root Mean Square Residual, *RMSEA* Root Mean Square Error of Approximation, *AIC* Akaike’s information criterion, *BIC* Bayesian Information Criterion.  ^g^Good value; ^a^acceptable value; ^n^unacceptable value.  *p < .05; **p < .01. | | | | | | | | | |

| **Table S3.** Confirmatory factor analyses for subsamples with different severity of depressive symptoms and invariance testing | | | | | | | | | | |
| --- | --- | --- | --- | --- | --- | --- | --- | --- | --- | --- |
|  | χ^2^ | *df* | χ^2^/*df* | Δχ^2^ (Δ*df*) | CFI | ΔCFI | SRMR | ΔSRMR | RMSEA | ΔRMSEA |
| Sample |  |  |  |  |  |  |  |  |  |  |
| Minimal/mild depressive symptoms (*n* = 119) | 7.95 | 4 | 1.99^g^ |  | .979^g^ |  | .041^g^ |  | .104^n^ |  |
| Moderate/severe depressive symptoms (*n* = 162) | 5.91 | 4 | 1.48^g^ |  | .989^g^ |  | .036^g^ |  | .054^a^ |  |
| Invariance level |  |  |  |  |  |  |  |  |  |  |
| Configural | 13.93 | 8 | 1.74 |  | .985 |  | .033 |  | .081 |  |
| Weak | 28.96** | 12 | 2.41 | 15.03 (4) | .961 | -.024 | .063 | .030 | .106 | .025 |
| Strong | 48.30*** | 16 | 3.02 | 19. 34(4) | .906 | -.055 | .102 | .039 | .126 | .020 |
| Note*.* χ^2^ = chi-square; df = Degrees of Freedom; Δχ^2^ = chi-square difference; CFI = Comparative Fit Index; ΔCFI = Change in CFI when compared to the baseline model; SRMR = Standardized Root Mean Square Residual; ΔSRMR = Change in SRMR when compared to the baseline model; RMSEA = Root Mean Square Error of Approximation; ΔRMSEA = Change in RMSEA when compared to the baseline model.  ^g^Good value; ^a^acceptable value; ^n^unacceptable value.  **p < .01; ***p < .001. | | | | | | | | | | |

| **Table S4** Spearman rho correlations between WHODAS and WSAS items | | | | | |
| --- | --- | --- | --- | --- | --- |
| Measures | WSAS 1 | WSAS 2 | WSAS 3 | WSAS 4 | WSAS 5 |
| WSAS 1. Work | 1 |  |  |  |  |
| WSAS 2. Home Management | .64^**^ | 1 |  |  |  |
| WSAS 3. Social Leisure | .54^**^ | .74^**^ | 1 |  |  |
| WSAS 4. Private Leisure | .51^**^ | .73^**^ | .72^**^ | 1 |  |
| WSAS 5. Family and Relationships | .41^**^ | .62^**^ | .70^**^ | .61^**^ | 1 |
| WHODAS 1. Standing | .17^**^ | .31^**^ | .24^**^ | .27^**^ | .25^**^ |
| WHODAS 2. Household | .39^**^ | .66^**^ | .47^**^ | .51^**^ | .39^**^ |
| WHODAS 3. Learning a new task | .40^**^ | .46^**^ | .50^**^ | .41^**^ | .45^**^ |
| WHODAS 4. Community activities | .33^**^ | .39^**^ | .52^**^ | .38^**^ | .42^**^ |
| WHODAS 5. Emotionally affected | .45^**^ | .46^**^ | .46^**^ | .43^**^ | .37^**^ |
| WHODAS 6. Concentrating | .33^**^ | .37^**^ | .41^**^ | .36^**^ | .37^**^ |
| WHODAS 7. Walking | .20^**^ | .31^**^ | .30^**^ | .28^**^ | .25^**^ |
| WHODAS 8. Body hygiene | .23^**^ | .28^**^ | .24^**^ | .23^**^ | .22^**^ |
| WHODAS 9. Getting dressed | .22^**^ | .25^**^ | .21^**^ | .25^**^ | .18^**^ |
| WHODAS 10. Dealing with new people | .32^**^ | .40^**^ | .47^**^ | .36^**^ | .45^**^ |
| WHODAS 11. Maintaining friendships | .26^**^ | .39^**^ | .52^**^ | .36^**^ | .48^**^ |
| WHODAS 12. Work | .50^**^ | .36^**^ | .34^**^ | .33^**^ | .30^**^ |
| Note. Sample size ranged from n = 269 to n = 277. **p < .001. *WSAS* Work and Social Adjustment Scale; *WHODAS* World Health Organization Disability Assessment Schedule. | | | | | |

| **Table S5** WSAS total score among patients with different severity of depressive symptoms | | | | |
| --- | --- | --- | --- | --- |
| Depressive symptoms (BDI-II)^a^ | N | Mean | SD | P value |
| Minimal | 54 | 6.31 | 5.71 | p < .001 |
| Mild | 62 | 14.40 | 6.43 |  |
| Moderate | 81 | 20.95 | 8.05 |  |
| Severe | 80 | 27.60 | 6.08 |  |
| Note. ^a^Kruskal-Wallis test*. BDI-II* Beck Depression Inventory II. | | | | |
